# Neuropeptide Y in the medial habenula alleviates migraine-like behaviors through the Y1 receptor

**DOI:** 10.1186/s10194-023-01596-z

**Published:** 2023-05-25

**Authors:** Chunxiao Yang, Zihua Gong, Xiaochen Zhang, Shuai Miao, Bozhi Li, Wei Xie, Tao Wang, Xun Han, Liang Wang, Zhao Dong, Shengyuan Yu

**Affiliations:** 1 School of Medicine, Nankai University, Tianjin, 300071 China; 2 Department of Neurology, the First Medical Center, Chinese PLA General Hospital, Fuxing Road 28, Haidian District, Beijing, 100853 China; 3 Medical School of Chinese PLA, Beijing, 100853 China; 4 Department of Medical Oncology, 980th Hospital of PLA Joint Logistical Support Force (Bethune International Peace Hospital), Shijiazhuang, Hebei 050082 China; 5 Academy of Medical Engineering and Translational Medicine, Tianjin University, Tianjin, 300072 China; 6 Institute of Neuroscience, Zhejiang University School of Medicine, Hangzhou, 310058 Zhejiang China

**Keywords:** Migraine, Glyceryl trinitrate, Neuropeptide Y, Y1 receptor, Medial habenula

## Abstract

**Background:**

Migraine is a highly disabling health burden with multiple symptoms; however, it remains undertreated because of an inadequate understanding of its neural mechanisms. Neuropeptide Y (NPY) has been demonstrated to be involved in the modulation of pain and emotion, and may play a role in migraine pathophysiology. Changes in NPY levels have been found in patients with migraine, but whether and how these changes contribute to migraine is unknown. Therefore, the purpose of this study was to investigate the role of NPY in migraine-like phenotypes.

**Methods:**

Here, we used intraperitoneal injection of glyceryl trinitrate (GTN, 10 mg/kg) as a migraine mouse model, which was verified by light-aversive test, von Frey test, and elevated plus maze test. We then performed whole-brain imaging with NPY-GFP mice to explore the critical regions where NPY was changed by GTN treatment. Next, we microinjected NPY into the medial habenula (MHb), and further infused Y1 or Y2 receptor agonists into the MHb, respectively, to detect the effects of NPY in GTN-induced migraine-like behaviors.

**Results:**

GTN effectively triggered allodynia, photophobia, and anxiety-like behaviors in mice. After that, we found a decreased level of GFP^+^ cells in the MHb of GTN-treated mice. Microinjection of NPY attenuated GTN-induced allodynia and anxiety without affecting photophobia. Furthermore, we found that activation of Y1—but not Y2—receptors attenuated GTN-induced allodynia and anxiety.

**Conclusions:**

Taken together, our data support that the NPY signaling in the MHb produces analgesic and anxiolytic effects through the Y1 receptor. These findings may provide new insights into novel therapeutic targets for the treatment of migraine.

**Graphical Abstract:**

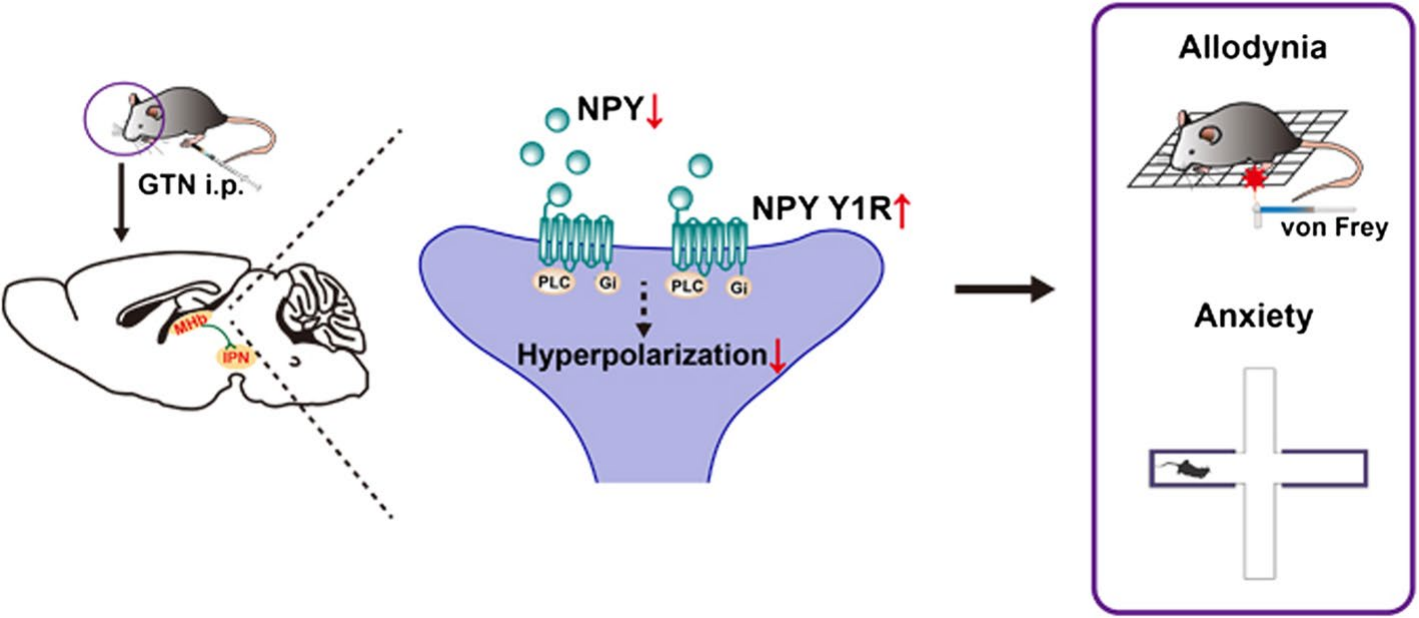

**Supplementary Information:**

The online version contains supplementary material available at 10.1186/s10194-023-01596-z.

## Introduction

Migraine is a complex, widespread, and paroxysmal neurologic disorder, affecting ~ 15% of the global population [1]. During the COVID-19 pandemic, a worsening of migraines was found in more than half of migraineurs [2]. Migraine is punctuated by a broad spectrum of sensory dysfunctions apart from headaches—including photophobia, phonophobia, osmophobia, and cutaneous allodynia [3]. In addition to pan-sensory gain, nausea and vomiting are prominent features, and patients can be physically, mentally, and socially incapacitated for days by migraine attacks [4]. Moreover, migraine is often associated with comorbid diseases, including epilepsy [5], depression and anxiety [6, 7], stroke [8], and other conditions [9]. These characteristics have led migraine to emerge as the second leading cause of disability [10]. Although there are various acute and prophylactic therapies for migraine, most focus only on headache-related symptoms while failing to address multisensory integration. Besides, currently available treatments are not effective in all patients with migraine [11]. Therefore, there is a clear and unmet need for additional refining of migraine management. Recently, neuropeptides and their receptors have gained much attention as novel treatments because of their proposed role in migraine pathogenesis [12]. Among them, neuropeptide Y (NPY) is a strong potential candidate.

The 36–amino acid peptide, NPY, is widely distributed throughout the central and peripheral nervous systems and many other tissues. Of these, the brain is the most abundant site of NPY expression [13]. NPY has been considered to play an important role in numerous physiological processes, including food intake [14], pain processing [15], emotion regulating [16] and modulation of cortical excitability [17]. All of these processes are known to be involved in migraine, suggesting a key role of NPY in migraine pathophysiology. The association between NPY levels and migraine has been extensively studied; however, no conclusions were reached. For example, Gallai et al. [18] reported that plasma NPY levels were decreased during the interictal period in juveniles with migraine, but increased during migraine attacks only in patients who experienced migraine with aura. In contrast, Siva et al. [19] showed higher serum NPY level in migraineurs during an attack-free period, and Goadsby et al. [20] showed that NPY levels in the external jugular venous blood remained unchanged during migraine attacks. Similar conflicting results were also found in cerebrospinal fluid [21, 22]. Considering the widespread expression of NPY in the brain, a deeper understanding of its variations in brain areas and its specific role in migraine is urgently needed.

NPY signals through four Y receptors in humans (Y1, Y2, Y4, and Y5) and Y6 in mice, all of which are expressed in the brain [23]. Naveilhan and colleagues [24] found that Y1-receptor-knockout mice displayed hyperalgesia and abolished the antinociceptive effects of NPY delivered intrathecally. Another study found that peripheral administration of NPY promoted pain via the Y2 receptor [25], suggesting a biphasic role of NPY in nociception at different sites with different receptors. Unfortunately, potential relationships between NPY levels and migraine remain uncertain. In addition, the Y5 receptor has been implicated in food intake [26] and hunger-dependent odor preferences [27]. While the Y4-receptor mRNA is the least-common NPY receptor subtype, it exhibits a much higher affinity for the pancreatic polypeptide than for NPY [28]. These data lead us to question the possible role of NPY in migraine and which type of receptors are involved.

It is well accepted that systemic administration of glyceryl trinitrate (GTN, a nitric oxide donor) produces migraine-like symptoms in animals, similar to effects observed in humans, and sensitizes structures that underlie migraine pathophysiology [29, 30]. Thus, we used GTN to model migraine in mice and investigated the role of NPY in migraine-like phenotypes. Using a brain-wide mapping of NPY expression with NPY-GFP mice, we identified that NPY levels in the medial habenula (MHb) were significantly decreased in GTN mice. Furthermore, site-directed infusion of NPY and a Y1 receptor agonist alleviated GTN-induced allodynia and anxiety-like behaviors. Our data provide the first evidence that NPY signaling through the Y1 receptor in the MHb critically modulates migraine-like behaviors.

## Methods

### Animals

Male mice were used throughout the study to eliminate possible hormone cycle effects. SPF-grade wild-type (C57BL/6 J) mice (8–10 weeks old) purchased from SiPeiFu Biotechnology Co., Ltd (Beijing, China) were used for the phenotype experiments and immunofluorescence staining. NPY-GFP transgenic mice (JAX stock #006417) in which GFP is expressed from the *Npy* promoter [31] were used for VISoR imaging.

The mice were group housed under tightly controlled temperature (21℃–24℃), humidity (40%–60%), and illumination conditions (12-h light/dark cycle, lights on at 9:00 AM) and with ad libitum access to food and water. All experimental procedures were approved by the Institutional Animal Care and Use Committee of the Chinese People’s Liberation Army (PLA) General Hospital, and animal experiments were conducted following the Animal Research: Reporting of in Vivo Experiments (ARRIVE) guidelines [32].

### Glyceryl trinitrate (GTN) mouse model of migraine

Glyceryl trinitrate (GTN) is the most common and well-established experimental migraine trigger in humans and rodents [33]. GTN induces migraine-like attacks with allodynia and conditioned place aversion [34]. We administered GTN to investigate potential mechanisms associated with migraine headaches. Mice were randomly assigned to either a GTN or vehicle (VEH) group. Experimenters were blind to group allocation. GTN (5 mg/ml in ethanol, Beijing Yimin Pharmaceutical Co., Ltd., Beijing, China) was freshly diluted in 0.9% (wt/vol) saline to a final concentration of 0.5 mg/ml for a dose of 10 mg/kg [35]. The vehicle containing 10% (vol/vol) alcohol was administered intraperitoneally in a 0.2 ml/10 g volume as a control. After administration, the mice were allowed to recover for at least 30 min in their home cages.

### Cannula implantation and drug microinjection

For stereotaxic surgery, the mice were deeply anesthetized by intraperitoneal injection (0.2 ml/10 g body weight) of 1.25% Avertin (a mixture of 12.5 mg/mL of 2,2,2-Tribromoethanol and 25 μl/mL 2-Methyl-2-butanol, Sigma, T48402, 152463, St. Louis, MO, USA), and then placed into a stereotaxic device (RWD Life Science, 69105, Shenzhen, China). After exposing the skull by scalp incision, bilateral craniotomies were created using a dental drill with 0.5-mm burs. Next, guide cannulas (27 gauge, 3.5 mm pedestal, RWD Life Science, 62004, Shenzhen, China) were implanted into the bilateral MHb (20˚ angle at the coordinates anteroposterior [AP] − 1.34 mm, mediolateral [ML] ± 1.21 mm, and dorsoventral [DV] − 2.70 mm), according to the Paxinos & Franklin Mouse Brain Atlas [36]. The cannulas were secured to the skull with screws and dental cement, and the stylets were inserted to avoid obstruction of the cannulas. The mice were allowed to recover from surgery for 10 days before behavioral testing.

During drug infusion, mice were maintained under light isoflurane (1%–1.5%) anesthesia, the stylets were removed and injection cannulas (33 gauge, RWD Life Science, 62204, Shenzhen, China) were inserted 0.5 mm beyond the guide cannulas for a final distance of 2.75 mm from the brain’s surface. The injection cannula was connected to a 10 μl microsyringe (Hamilton, Nevada, USA) via a polyethylene tube driven by a syringe pump (KD Scientific, 788130, USA). NPY (2 μg/μl in saline, Tocris Bioscience, 1153, UK), Y1R agonist ([Leu31,Pro34]-NPY, 2 μg/μl in saline, MedChemExpress, HY-P1323A, Monmouth Junction, USA), Y2R agonist (Peptide YY, 2 μg/μl in saline, MedChemExpress, HY-P1021A, Monmouth Junction, USA) or saline were infused bilaterally into the MHb at a flow rate of 100 nl/min (200 nl/side). The intracranial injection concentrations were determined based on previous studies [37, 38]. After injection, the cannulas were left in place for 5 min to allow for drug diffusion. Subsequently, the injection cannulas were removed and the stylets were re-inserted. Drug injections were performed immediately before induction of the GTN migraine mouse model. Cannula placements were verified postmortem by infusing 200 nl of 0.25% Evans Blue (Sigma, E2129, St. Louis, MO, USA) into the MHb before sacrifice, and were imaged at 10 × objective on an optical microscope (Olympus, DP73, Tokyo, Japan). Only data from properly injected mice were used for statistical analyses.

### Light-aversive test

**Fig. 1:**
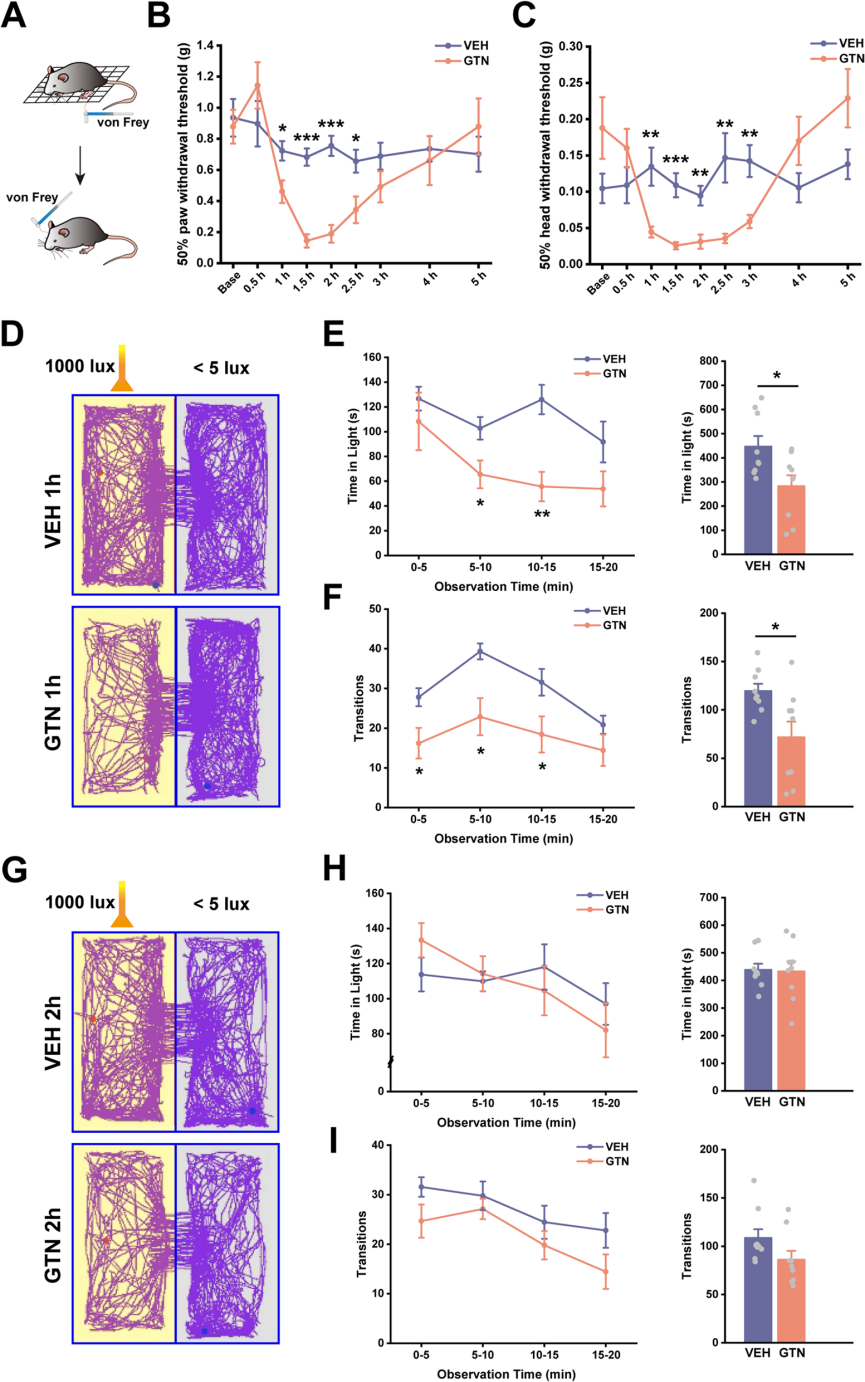
Acute glyceryl trinitrate (GTN) administration produces transient allodynia and photophobia. **A** The schematic illustration of the von Frey test indicates the region where the von Frey stimulus was applied during testing. **B** and **C** Time course of GTN-induced changes in left hind paw (**B**) and head withdrawal thresholds (**C**) based on von Frey tests. **D** and **G** Example traces of mice in 20-min light-aversive test at 1 h (**D**) and 2 h (**G**) after VEH or GTN injection. **E** and **H** Average time spent in light for each group per 5-min interval (left panel) and over a total of 20 min (right panel) at 1 h (**E**) and 2 h (**H**) after VEH or GTN injection. **F** and **I** The number of transitions between zones expressed as the average for each group per 5-min interval (left panel) and during a total of 20 min (right panel) at 1 h (**F**) and 2 h (**I**) after VEH or GTN injection. *n* = 9 mice per group. Significance was assessed by two-way repeated-measures ANOVA with post hoc comparison between groups (B, C, E left panel, F left panel, H left panel, and I left panel) or by two-tailed unpaired Student’s t-test (E right panel, F right panel, H right panel, and I right panel). All data are presented as the mean ± S.E.M. *P < 0.05, **P < 0.01, ***P < 0.001

For all behavioral testing, mice were acclimatized in their cages in the testing room for at least 2 h. To evaluate the typical migraine-associated photophobia induced by GTN, we used a modified light/dark box with infrared beam tracking (Shanghai Xinruan Information Technology Co., Ltd., XR-XB120, Shanghai, China). The box (30 cm wide × 30 cm deep × 30 cm high) consisted of two equally sized compartments: one painted white and brightly lit (1000 lx) with an LED panel, the other painted black and not lit (< 5 lx). A corridor (7 cm × 7 cm) connected the two compartments and allowed the mice to move freely. Light-aversive behaviors were examined in different batches of mice separately during the early (50–70 min) and late phases (110–130 min) after GTN/VEH injection (Fig. 1), according to previous studies [39, 40]. Each mouse was gently placed in the center of the light zone, facing away from the dark side. Between animals, the box was thoroughly cleaned using 75% alcohol.

All data were recorded and analyzed using a SuperMaze video-tracking system (Shanghai Xinruan Information Technology Co., Ltd., XR-Xmaze, Shanghai, China). We excluded mice who were inactive for 90% or more of the testing time. Decreased transitions and time spent in light were considered to reflect light-aversion behaviors.

### von Frey Test

Cutaneous allodynia is a form of sensory amplification that occurs during a migraine attack. Consequently, facial and hind paw mechanical allodynia were quantified using calibrated von Frey filaments (Aesthesio®, Danmic Global, San Jose, CA, USA). To test head thresholds, the hair on the mice’s foreheads (above and between two eyes) was shaved, and mice were handled extensively for at least 3 days before testing for 5 min per day. On the test day, the experimenter gently held the mouse on the palm with minimal restraint and a series of calibrated von Frey filaments were perpendicularly applied to the shaved skin, causing the filaments to bend for 3 s. As previously described [41], criteria for a positive response included the mouse vigorously stroked face with the forepaw, head withdrawal, or head shaking. For hind paw testing, mice were placed in plexiglass chambers (10 cm wide × 7 cm deep × 16 cm high), one mouse per chamber, on a mesh floor to acclimate for 30 min during each of the 3 days before testing. The plantar surface of the left hind paw was stimulated with von Frey filaments until a withdrawal of the paw or paw licking occurred.

We used the up-down method [42], where, if the animal produced a negative response, the researchers applied the filament with the next-greater force. If the animal produced a positive response, the stimulus was decreased. After the first breaking point (change in response), four more stimulations were applied, the response pattern and final filament were noted, and 50% withdrawal thresholds were calculated using a freely available online algorithm at https://bioapps.shinyapps.io/von_frey_app/ [43].

We determined the 50% hind paw and head withdrawal thresholds before injection (baseline) and 0.5, 1, 1.5, 2, 2.5, 3, 4, and 5 h after GTN/VEH injection in Fig. 1.

### Elevated Plus Maze (EPM) test

Anxiety-like behaviors related to migraine were evaluated using an elevated plus maze (EPM; Shanghai Xinruan Information Technology Co., Ltd., XR-XG201, Shanghai, China). The EPM consisted of two open (35 cm wide × 5 cm deep, 60 lx) and two closed arms (35 cm wide × 5 cm deep × 15 cm high, < 5 lx) connected by a central platform (5 cm wide × 5 cm deep), forming a plus. The maze was elevated 60 cm from the floor. Mice were placed in the central platform facing an open arm and were allowed to freely explore the EPM for 10 min. The maze was thoroughly cleaned with 75% alcohol between mice. Movements were tracked and analyzed with SuperMaze software (Shanghai Xinruan Information Technology Co., Ltd., XR-Xmaze, Shanghai, China). Time spent in open/closed arms and the number of open/closed-arm entries were determined by the software.

**Fig. 2:**
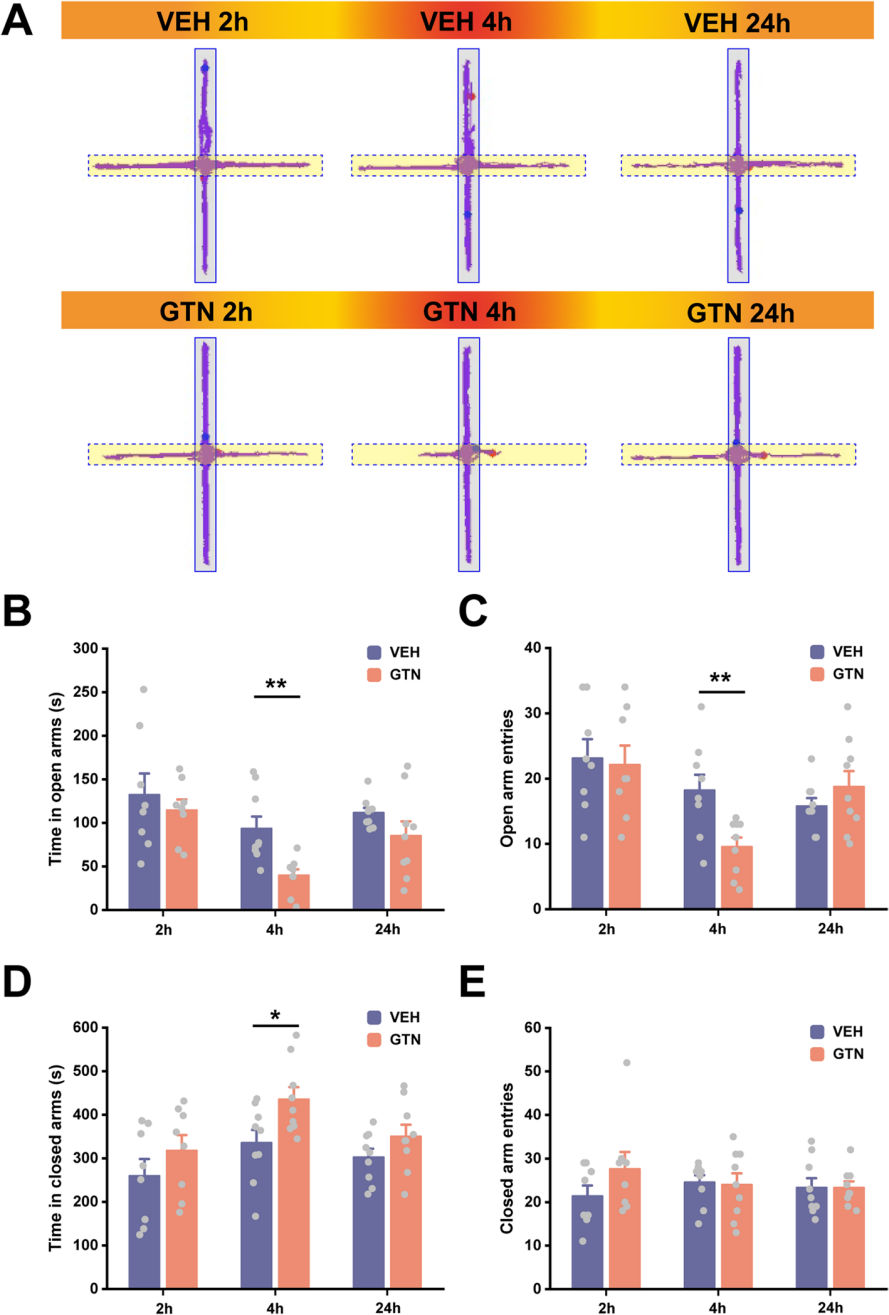
Acute glyceryl trinitrate (GTN) administration induces delayed occurrence of anxiety-like behaviors. **A** Representative traces of mice in 10-min Elevated Plus Maze (EPM) test at 2 h, 4 h, and 24 h after VEH or GTN injection. **B**-**E** Time spent in the open arms (**B**), the number of open arm entries (**C**), time spent in the closed arms (**D**), and the number of closed-arm entries (**E**) during the EPM test at 2 h (*n* = 8 mice per group), 4 h (*n* = 9 mice per group), and 24 h (*n* = 9 mice per group) after VEH or GTN injection. Significance was assessed by a two-tailed unpaired Student’s t-test. All data are presented as the mean ± S.E.M. *P < 0.05, **P < 0.01

EPM test was performed in different batches of mice separately at 2, 4, and 24 h after GTN/VEH injection in Fig. 2.

### Whole-brain imaging and image processing

Since NPY is widely distributed throughout the central nervous system, we performed whole-brain mapping of NPY expression in NPY-GFP mice with high-speed and high-throughput Volumetric Imaging with Synchronized on-the-fly-scan and Readout (VISoR), as previously described [44]. Two hours after intraperitoneal (i.p.) injection of GTN or VEH, the mice were deeply anesthetized with 1.5% Avertin and transcardially perfused with 20 ml of 0.1 M phosphate-buffered saline (PBS, pH 7.4) at 37 °C and 4 °C, respectively, followed by 20 ml of ice-cold 4% (wt/vol) paraformaldehyde (PFA) in PBS. Then brains were carefully removed and post-fixed in 4% PFA for 24 h at 4℃. Next, the brains were transferred into 4% hydrogel monomer solution (HMS, 4% acrylamide, 0.05% bis-acrylamide, 4% PFA, and 0.25% VA-044 thermal initiator in PBS (wt/vol)) for 2 days at 4 °C, allowing penetration of fixation solution. Then, the samples were embedded in an equal volume mixture of 20% bovine albumin serum and 4% HMS for 4 h at 37 °C, followed by three washes (2 h each) with room-temperature PBS.

The embedded samples were sectioned into 300-μm consecutive coronal slices using a vibrating microtome (Compresstome VF-300, Precisionary Instruments, Greenville, NC, USA). The brain slices were cleared in 5% (vol/vol) PBST (5% Triton X-100 in PBS) with gentle shaking for 24 h at 37 °C, then rinsed in PBS three times (8 h each).

Lastly, the brain slices were mounted on glass slides in sequence and were polymerized with 4% HMS for 4 h at 37 °C. After three rinses with PBS, the slides were immersed overnight in a refractive-index-matching solution (80% iohexol in PBS (wt/vol)) with a refractive index of 1.52.

All slices were imaged with a custom VISoR microscope at 1 × 1 × 2.5 μm^3^ voxel resolution and stitched together to automatically reconstruct the whole mouse brain volume using custom-developed algorithms and software [44]. To identify the specific brain regions, the images were down sampled to 4 × 4 × 4 μm^3^ resolution and registered to the Allen Mouse Brain Common Coordinate Framework (Allen CCF) [45] using custom software.

For NPY cell counting, the images of 4 × 4 × 4 μm^3^ resolution were stacked to images of 25 µm in the Z direction with ImageJ. Cell counting was first trained manually to identify and count the neurons within 40 representative, 25-µm images using Ilastik software (version 1.3.3post3, University of Heidelberg, Heidelberg, Germany). Then, all of these 25-µm images were automatically processed in batches. Finally, we matched the positions of NPY neurons to the Allen CCF using custom software to determine the number of neurons within each brain area.

### Immunofluorescence staining and imaging

To evaluate the expression levels of NPY Y1 receptor and Y2 receptor in the MHb, we performed immunofluorescence staining in C57BL/6 J mice. The perfusion process was the same as that for VISoR imaging. After post-fixation, brains were subsequently infiltrated with 15% (wt/vol) and 30% (wt/vol) sucrose sequentially until they sank to the bottom, embedded in O.C.T. TissueTek Compound (Sakura Finetek, 4583, Torrance, CA, USA), and then sectioned into 30-μm coronal slices using a freezing microtome (Leica Biosystems, CM1950, Heidelberger, Germany), after which they were stored in PBS. Free-floating sections were incubated with a blocking buffer containing 10% (vol/vol) normal donkey serum and 0.25% (vol/vol) Triton X-100 dissolved in PBS for 1.5 h at room-temperature. The sections were then incubated overnight at 4℃ with primary rabbit anti-Y1 receptor (1: 500, Neuromics, RA24506, Edina, MN, USA) or rabbit anti-Y2 receptor (1: 500, Neuromics, RA14112, Edina, MN, USA) diluted in blocking buffer. Next, sections were washed 3 times (20 min each) with 0.25% PBST (0.25% Triton X-100 in PBS) and incubated with a fluorescent dye-conjugated donkey anti-rabbit secondary antibody (1:1000, Abcam, ab150061, Waltham, MA, USA) for 2 h at room-temperature. Following three washes (20 min each) with PBST, sections were mounted and coverslipped with mounting medium with DAPI (Abcam, ab104139, Waltham, MA, USA).

Fluorescence images were acquired as z-stacks (2 µm step size for 20 µm per stack) using a pannoramic confocal scanner (3DHISTECH Ltd., Budapest, Hungary) with a 20 ×, 0.8 NA objective. Six sections were selected from the MHb (from 1.06–2.06 mm posterior to bregma) for each receptor from an individual animal. Intensity quantifications were performed using ImageJ software (Fiji, NIH, USA). Based on reports in related literature, the sample size was not predetermined by calculation.

### Statistical analysis

All experiments and data analyses were conducted by experimenters blinded to group assignment. Sample sizes were determined by previous experience, and all were numbers of biological repeats. Data are shown as mean ± standard error of the mean (S.E.M.). Two-tailed unpaired Student’s t-test, one-way ANOVA, two-way ANOVA, two-way repeated-measures ANOVA, and Kruskal–Wallis H test were performed using SPSS version 22 (IBM Analytics, Armonk, NY, USA). Details of each analysis are included in the figure legends and statistical significance was set at *P* < 0.05.

## Results

### Phenotypic characterization of the GTN-induced migraine model in mice

Although the GTN mouse model is well validated by its induction of migraine-like headaches and response to specific migraine drugs [46, 47], researchers rarely investigated non-headache symptoms in a time-dependent manner. To investigate the impact of GTN on both sensory and affective aspects of migraine-like behaviors, we treated male C57BL/6 J mice with GTN or vehicle (VEH) and tested them for generalized allodynia, photophobia, and anxiety-like behaviors. As shown in Fig. 1A–C, mice administered with GTN displayed a time-dependent decrease in mechanical withdrawal thresholds of the hind paw (time × group interaction: *F*(3.490, 55.835) = 5.106, *P* = 0.002; simple effects: 1 h: *F*(1, 16) = 6.667, *P* = 0.020; 1.5 h: *F*(1, 16) = 52.122, *P* < 0.001; 2 h: *F*(1, 16) = 38.362, *P* < 0.001; 4 h: *F*(1, 16) = 0.161, *P* = 0.694) as well as the head (time × group interaction: *F*(4.143, 66.296) = 7.197, *P* < 0.001; simple effects: 1 h: *F*(1, 16) = 9.585, *P* = 0.007; 1.5 h: *F*(1, 16) = 20.567, *P* < 0.001; 2 h: *F*(1, 16) = 12.829, *P* = 0.002; 4 h: *F*(1, 16) = 2.435, *P* = 0.138) relative to VEH-treated mice. These effects developed within 1 h and reached their lowest levels at 1.5 h. Mechanical hypersensitivity had recovered gradually at 2 h, then subsided at 4 h after GTN administration.

Besides allodynia, we evaluated light-aversive behaviors (photophobia) using a modified light/dark box. Within the first 50 min after GTN injection, the mice showed remarkable reductions in locomotor activity due to the cardiovascular effects (hypotension) of GTN [48]. Consequently, these mice were inactive for more than 90% of the time spent in the box (data not shown). Because of this, we performed the light-aversion test at 1 h (early phase) and 2 h (late phase) after GTN administration. The durations comprised four intervals of 5 min each. Firstly, in the early phases after drug injection, GTN mice spent significantly less time in the light compared with VEH mice during the total 20 min (Fig. 1E right panel; *t*(2, 16) = 2.633, *P* = 0.018), especially at the 5–10 min and 10–15 min intervals (Fig. 1E left panel; time × group interaction: *F*(1.800, 28.793) = 1.674, *P* = 0.207; time: *F*(1.800, 28.793) = 5.195, *P* = 0.014; group: *F*(1, 16) = 6.932, *P* = 0.018; 5–10 min interval: *F*(1, 16) = 5.865, *P* = 0.028; 10–15 min interval: *F*(1, 16) = 15.526, *P* = 0.001). We further analyzed the number of transitions between the light and dark chambers as an indicator of light-aversive behavior. Compared to VEH mice, GTN-treated mice transitioned less at 0–5 min, 5–10 min, and 10–15 min intervals (Fig. 1F left panel; time × group interaction: *F*(3, 48) = 1.549, *P* = 0.214; time: *F*(3, 48) = 11.402, *P* < 0.001; group: *F*(1, 16) = 7.310, *P* = 0.016; 0–5 min interval: *F*(1, 16) = 5.909, *P* = 0.030; 5–10 min interval: *F*(1, 16) = 9.411, *P* = 0.011; 10–15 min interval: *F*(1, 16) = 4.774, *P* = 0.044) as well as during the total 20 min (Fig. 1F right panel; *t*(2, 11.159) = 2.704, *P* = 0.020). However, in the late phases, referred to as 2 h, GTN mice showed no signs of photophobia in both times in light and transitions (Fig. 1G–I; H left panel: time × group interaction:* F*(3, 48) = 1.049, *P* = 0.380; time: *F*(3, 48) = 3.166, *P* = 0.033; group: *F*(1, 16) = 0.012, *P* = 0.913; H right panel: *t*(2, 16) = 0.111, *P* = 0.913; I left panel: time × group interaction: *F*(3, 48) = 0.485, *P* = 0.694; time: *F*(3, 48) = 7.178, *P* < 0.001; group: *F*(1, 16) = 3.047, *P* = 0.100; I right panel: *t*(2, 16) = 1.746, *P* = 0.100).

Anxiety and mood disorders are the most frequent psychiatric migraine comorbidities [49]. Pre-clinical studies reported anxiety-like behaviors, mainly in chronic migraine animal models [50, 51]. In this study, we quantified the degree of anxiety in an acute migraine mouse model induced by GTN with an EPM test. We found that a single GTN injection reduced the time spent in open arms and the number of open arm entries (Fig. 2A–C; B: *t*(2, 11.797) = 3.477, *P* = 0.005; C: *t*(2, 16) = 3.142, *P* = 0.006). Meanwhile, time in closed arms increased (Fig. 2D; *t*(2, 16) =  − 2.466, *P* = 0.025) at 4 h after GTN application. Interestingly, no differences were observed at 2 h or 24 h (Fig. 2A–E), suggesting delayed anxiety after recovering from mechanical allodynia caused by GTN. Furthermore, there was no significant difference between the groups in the total distance travelled. This finding suggested that EPM test performance was not influenced by functional motor differences (Fig. S1). Taken together, these results demonstrated that acute GTN administration is an effective and reliable migraine model for triggering allodynia, photophobia, and anxiety-like behaviors in mice. We also determined the “best” timepoints for examination of different behaviors. More importantly, we revealed the evolutive sequence of symptoms induced by GTN. The observed sequences mimicked the symptom progression seen in patients who experience frequent migraine attacks.

### GTN alters NPY expression in the MHb

**Fig. 3:**
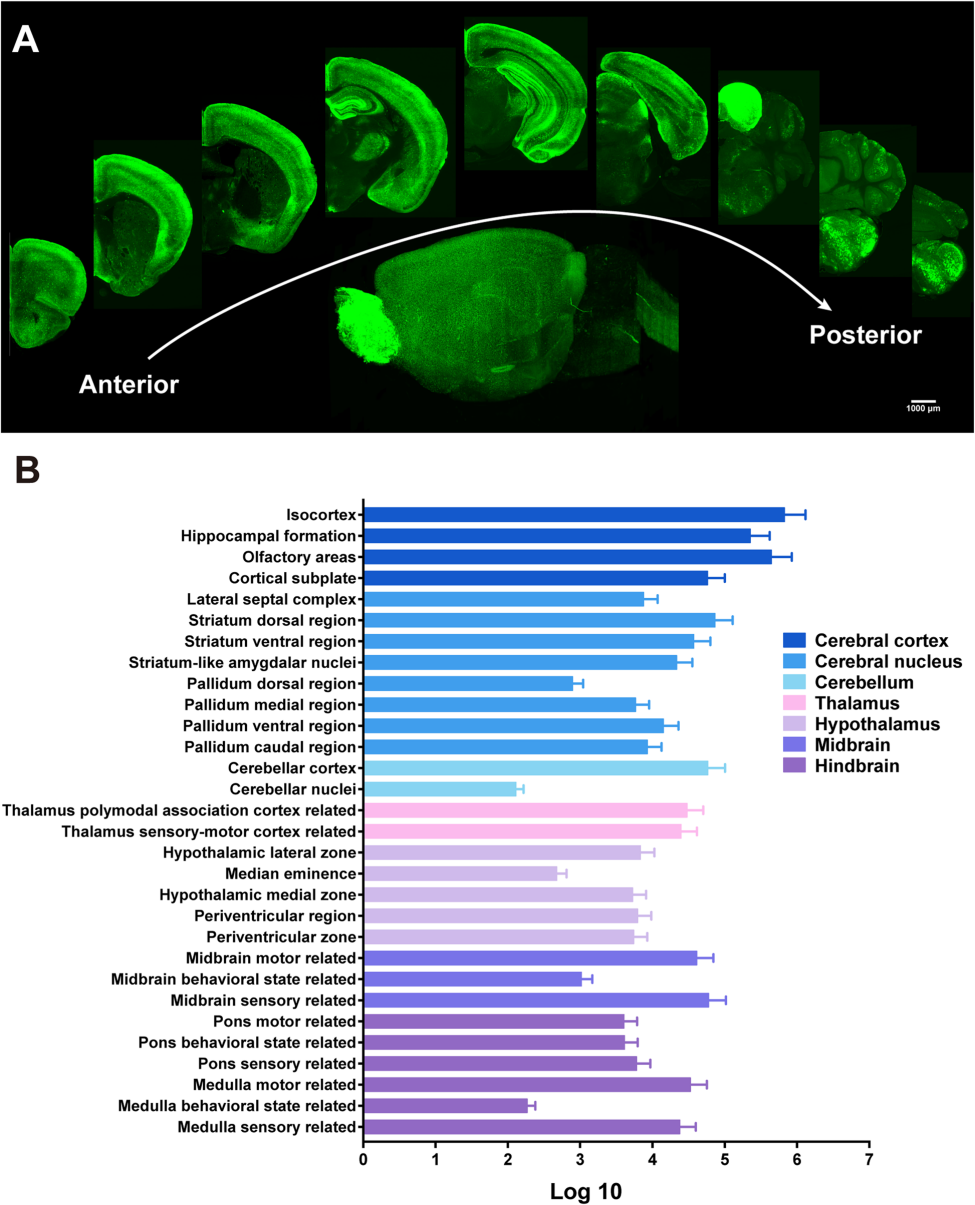
VISoR imaging of NPY-GFP mice shows the distribution of NPY across the whole brain. **A** The representative reconstructed NPY (green) images from a VEH mouse brain (scale bar, 1000 μm). **B** Quantitative NPY neurons across the whole-brain areas from the VEH group. *n* = 6 mice per area

NPY is widely distributed throughout the central nervous system. To identify the critical regions expressing NPY in the brain that may be responsible for the migraine-like phenotypes in the GTN mice, we used whole-brain VISoR imaging, as previously described by Wang et al*.* [44]. NPY-GFP reporter mice were subjected to GTN or VEH injection. Two hours later, brains from these mice were processed and imaged. The NPY levels were quantified using previously established counting methods. Representative images and the anatomic distribution of NPY neurons from VEH mice are shown in Fig. 3. Overall, NPY was highly expressed in the cerebral cortex (including neocortex, hippocampus, and olfactory bulb), striatum, thalamus (reticular nucleus of the thalamus, ventral posterior complex of the thalamus, habenula, and medial geniculate complex), hypothalamic nuclei (arcuate nucleus, paraventricular nucleus), superior colliculus sensory related, inferior colliculus, periaqueductal gray, and medulla (Fig. 3A, B).

**Fig. 4:**
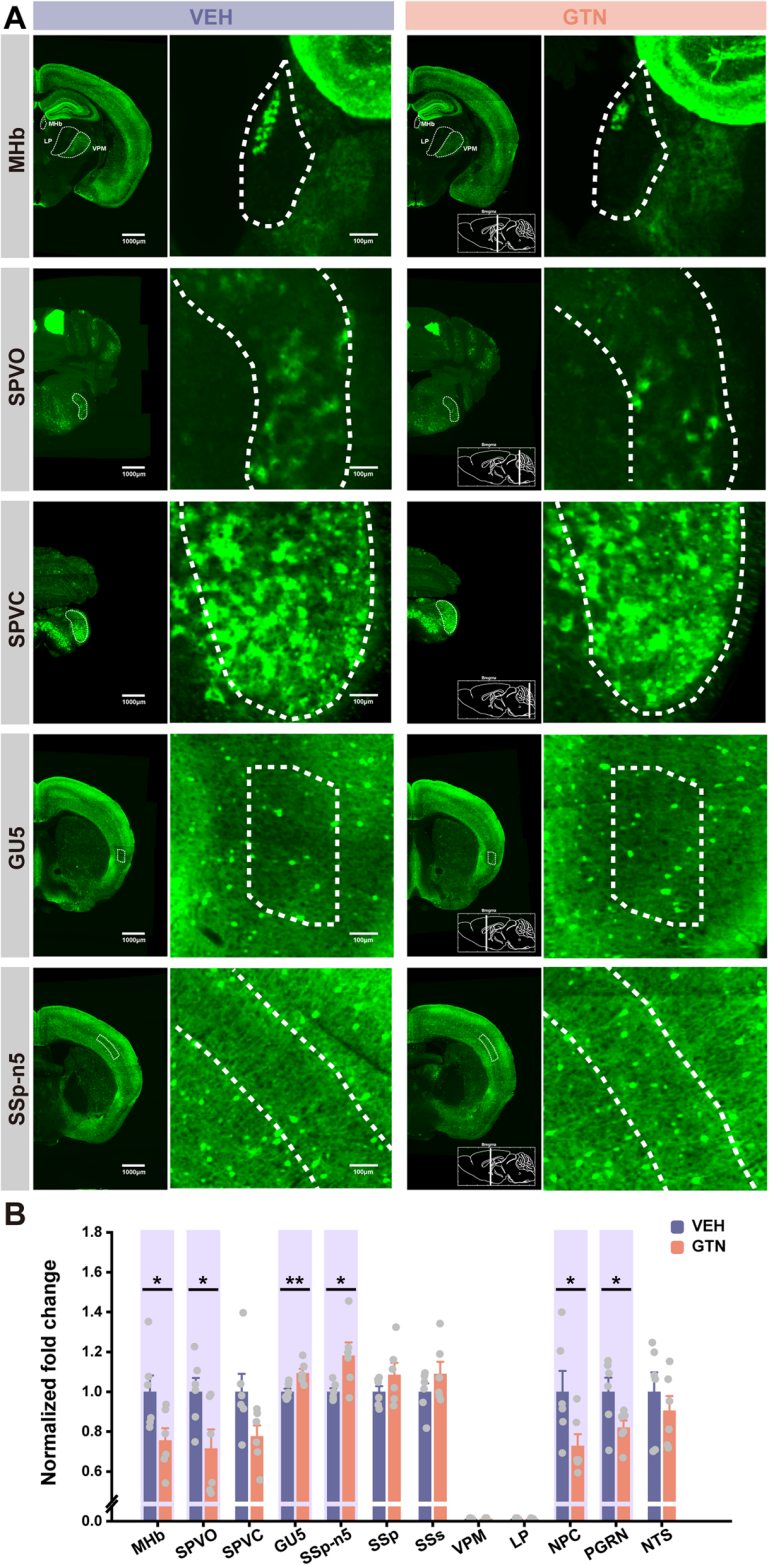
GTN alters NPY expression in several brain areas, including the medial habenula (MHb). **A** The representative reconstructed NPY (green) images from a VEH or GTN mouse brain in diverse brain areas. Each group's left panel is the overview (scale bar, 1000 μm), and higher magnification close-ups are shown in the right panel (scale bar, 100 μm). The white contour delineates the borders of the respective brain areas. **B** Summary fold changes in the number of NPY neurons of the GTN group over the VEH group in multiple brain areas. *n* = 6 mice per group. Significance was assessed using a two-tailed unpaired Student’s t-test. Data are presented as the mean ± S.E.M. *P < 0.05, **P < 0.01. MHb, medial habenula; SPVO, spinal nucleus of the trigeminal oral part; SPVC, spinal nucleus of the trigeminal caudal part; GU5, gustatory areas layer 5; SSp-n5, primary somatosensory area nose layer 5; SSp, primary somatosensory area; SSs, supplemental somatosensory area; VPM, ventral posteromedial nucleus of the thalamus; LP, lateral posterior nucleus of the thalamus; NPC, nucleus of the posterior commissure; PGRN, paragigantocellular reticular nucleus; NTS, nucleus of the solitary tract

While in response to GTN treatment, there were no significant differences in NPY neuron populations in some migraine-related brain areas, such as the spinal nucleus of the trigeminal caudal part (SPVC, *t*(2, 10) = 2.121, *P* = 0.060), ventral posteromedial nucleus of the thalamus (VPM), lateral posterior nucleus of the thalamus (LP), and nucleus of the solitary tract (NTS, *t*(2, 10) = 0.776, *P* = 0.456). Importantly, we noted a statistically significant decrease in GFP^+^ cells in certain brain areas, including the medial habenula (MHb, *t*(2, 10) = 2.376, *P* = 0.039), spinal nucleus of the trigeminal oral part (SPVO, *t*(2, 10) = 2.421, *P* = 0.036), nucleus of the posterior commissure (NPC, *t*(2, 10) = 2.261, *P* = 0.047), and paragigantocellular reticular nucleus (PGRN, *t*(2, 10) = 2.266, *P* = 0.047) (Fig. 4A, B). Oppositely, compared with the VEH group, more GFP^+^ cells were found in the gustatory areas layer 5 (GU5, *t*(2, 10) =  − 3.429, *P* = 0.006) and primary somatosensory area nose layer 5 (SSp-n5, *t*(2, 10) =  − 2.608, *P* = 0.026) in GTN mice (Fig. 4A, B). Among them, the MHb is a key site for pain modulation and affective and motivational processes [52]. Given that the NPY is also a potent neuromodulator playing roles in analgesia and antianxiety effects, changed NPY expression in the MHb by GTN might be the cause of GTN-induced migraine-like phenotypes. The numbers of NPY neurons in other brain areas from VEH and GTN mice were presented in Table S1.

### NPY signaling in the MHb alleviates GTN-induced allodynia and anxiety-like behaviors

**Fig. 5:**
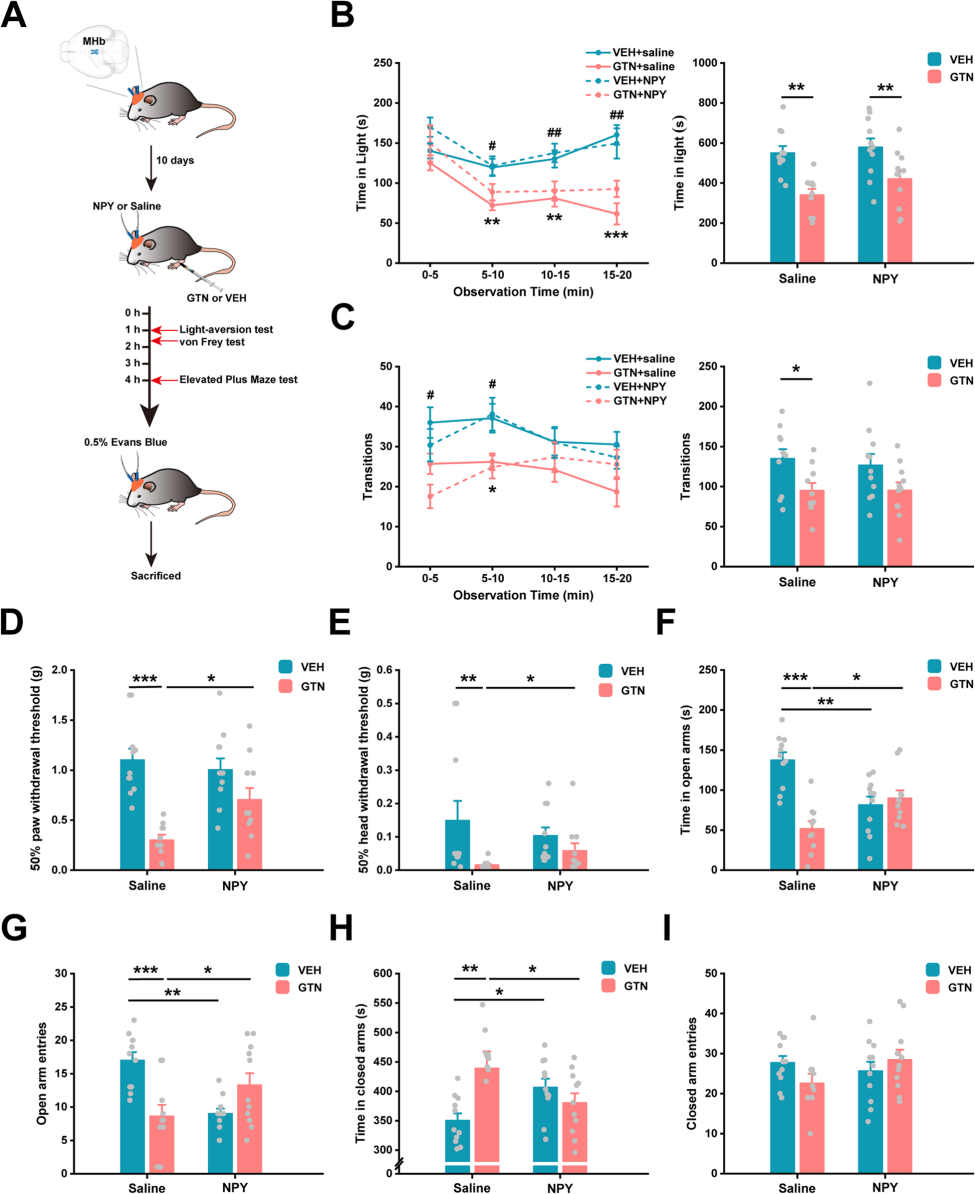
Microinjection of NPY into the MHb alleviates GTN-induced allodynia and anxiety-like behaviors. **A** Plan of the experimental procedure: MHb microinjections were performed immediately before VEH or GTN injection. **B** and **C** Effects of NPY or saline on time spent in light (**B**) and the number of transitions between zones (**C**) expressed as the average for each group per 5-min interval (left panel) and during a total of 20 min (right panel) in a light-aversive test at 1 h after VEH or GTN injection. **D** and **E** Effects of NPY or saline on the left hind paw (**D**) and head withdrawal thresholds (**E**) during the von Frey test at 1.5 h after VEH or GTN injection. **F**-**I** Effects of NPY or saline on time spent in the open arms (**F**), the number of open arm entries (**G**), time spent in the closed arms (**H**), and the number of closed arm entries (**I**) during the EPM test at 4 h after VEH or GTN injection. VEH + saline, *n* = 11 mice; GTN + saline, *n* = 10 mice; VEH + NPY, *n* = 11 mice; GTN + NPY, *n* = 11 mice. Significance was assessed by two-way repeated-measures ANOVA with post hoc comparison between groups (B and C left panel; *P < 0.05, **P < 0.01, ***P < 0.001 VEH + saline versus GTN + saline; #*P* < 0.05, ##*P* < 0.01 VEH + NPY versus GTN + NPY) or by two-way ANOVA with post hoc comparison between groups (B and C right panel, D, F-I; *P < 0.05, **P < 0.01, ***P < 0.001) or by Kruskal–Wallis H test with Mann–Whitney U post hoc comparison between groups (E; *P < 0.05, **P < 0.01). All data are presented as the mean ± S.E.M

To determine the role of MHb NPY in the development of allodynia, photophobia, and anxiety under GTN condition, we performed microinjection of NPY into the bilateral MHb immediately before GTN injection. Then, we sequentially tested light-aversive behaviors at 1 h, mechanical withdrawal thresholds at 1.5 h, and anxiety-like behaviors at 4 h after GTN injection. Control mice were injected with VEH + saline (Fig. 5A). A representative cannula location is shown in Fig. S4. Consistent with our results (Figs. 1 and 2), GTN + saline mice exhibited photophobia, allodynia, and anxiety-like behaviors compared to VEH + saline mice (Fig. 5B–H). However, NPY in the MHb had no influence on GTN-induced reductions in time-in-light and number of transitions in the light/dark box (Fig. 5B, C; B left panel: time × group interaction: *F*(9, 117) = 1.513, *P* = 0.151; time: *F*(3, 117) = 13.138, *P* < 0.001; group: *F*(3, 39) = 8.095, *P* < 0.001; 0–5 min interval: *F*(3, 39) = 1.437, *P* = 0.247; 5–10 min interval: *F*(3, 39) = 5.062, *P* = 0.005; 10–15 min interval: *F*(3, 39) = 5.662, *P* = 0.003; 15–20 min interval: *F*(3, 39) = 9.910, *P* < 0.001; B right panel: model × drug interaction: *F*(1, 39) = 0.456, *P* = 0.504; model: *F*(1, 39) = 22.352, *P* < 0.001; drug: *F*(1, 39) = 1.968, *P* = 0.169; C left panel: time × group interaction: *F*(9, 117) = 2.087, *P* = 0.036; simple effects: 0–5 min interval: *F*(3, 39) = 4.781, *P* = 0.006; 5–10 min interval: *F*(3, 39) = 4.065, *P* = 0.013; 10–15 min interval: *F*(3, 39) = 0.783, *P* = 0.511; 15–20 min interval: *F*(3, 39) = 1.975, *P* = 0.134; C right panel: model × drug interaction: *F*(1, 39) = 0.134, *P* = 0.716; model: *F*(1, 39) = 9.543, *P* = 0.004; drug: *F*(1, 39) = 0.106, *P* = 0.746). Strikingly, infusion of NPY robustly increased both hind paw (model × drug interaction: *F*(1, 39) = 5.739, *P* = 0.021; simple effects: *P* = 0.047, GTN + saline versus GTN + NPY) and head withdrawal thresholds (*χ*^*2*^(3) = 17.053, *P* = 0.001; *U* = 23.000, *P* = 0.024, GTN + saline versus GTN + NPY), time spent in open arms (model × drug interaction: *F*(1, 39) = 22.491, *P* < 0.001; simple effects: *P* = 0.010, GTN + saline versus GTN + NPY) and number of open arm entries (model × drug interaction: *F*(1, 39) = 19.663, *P* < 0.001; simple effects: *P* = 0.028, GTN + saline versus GTN + NPY) and also consistently reduced time in closed arms (model × drug interaction: *F*(1, 39) = 9.681, *P* = 0.003; simple effects: *P* = 0.032, GTN + saline versus GTN + NPY) in EPM compared to treatment with saline under the GTN paradigm (Fig. 5D–H). Unpredictably, VEH + NPY mice showed a significantly shorter time in open arms (model × drug interaction: *F*(1, 39) = 22.491, *P* < 0.001; simple effects: *P* = 0.001, VEH + saline versus VEH + NPY) and fewer number of open arm entries (model × drug interaction: *F*(1, 39) = 19.663, *P* < 0.001; simple effects: *P* = 0.001, VEH + saline versus VEH + NPY) in EPM, accompanied by a longer time in closed arms (model × drug interaction: *F*(1, 39) = 9.681, *P* = 0.003; simple effects: *P* = 0.035, VEH + saline versus VEH + NPY), when compared to VEH + saline mice (Fig. 5F-H). Plus, no difference was found between groups in the total distance travelled in EPM (Fig. S2). Taken together, NPY in the MHb attenuated GTN-induced allodynia and anxiety without affecting photophobia, whereas microinjection of NPY in the VEH mice led to an increase in anxiety-like behavior.

### NPY signaling in the MHb exerts its analgesic and anxiolytic effects through the Y1 receptor in GTN mice

**Fig. 6:**
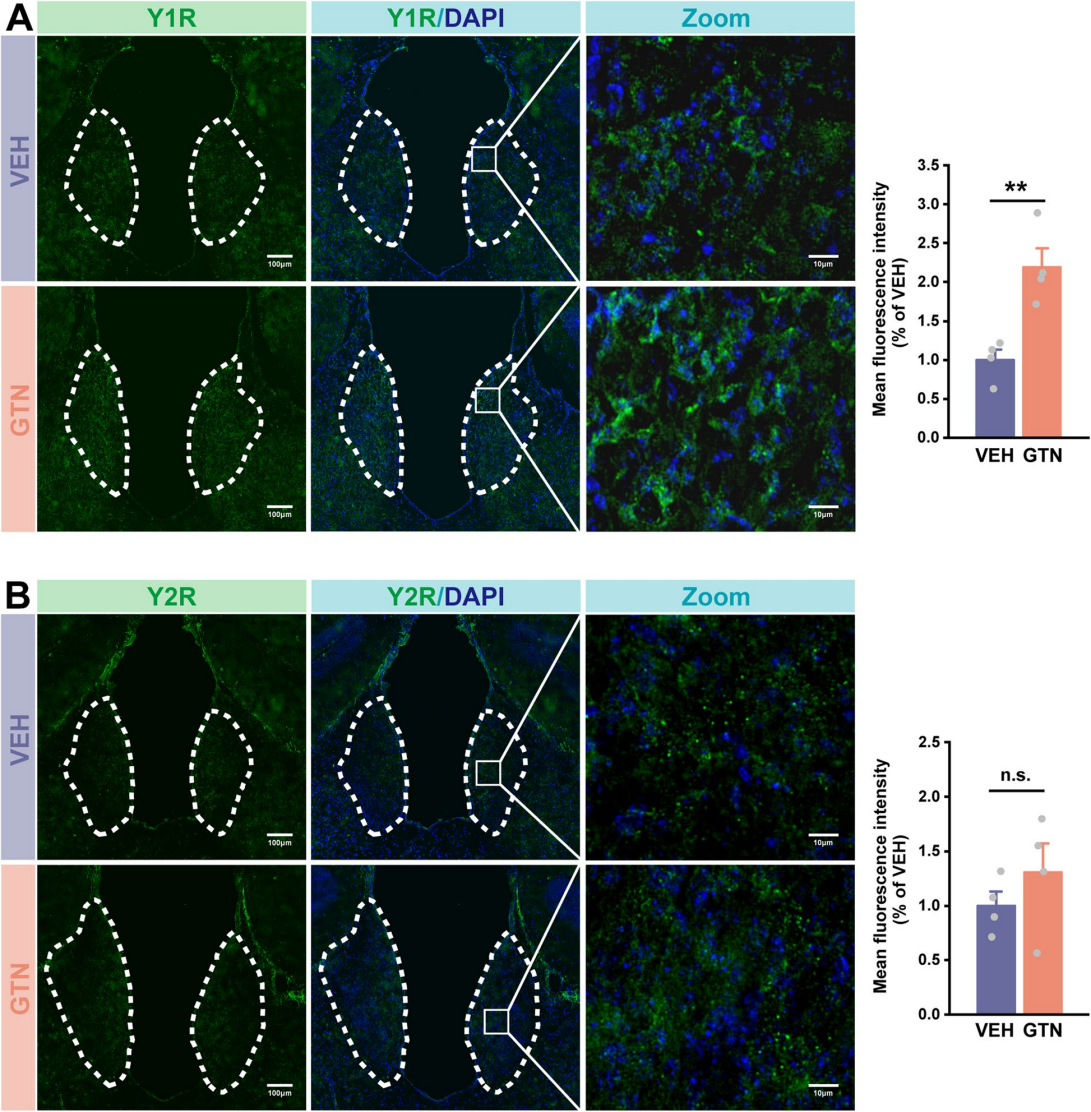
GTN increases the expression the of Y1 receptor in the MHb, but not affects the Y2 receptor. **A** and **B** Left: representative images of Y1 receptor (**A**, green), Y2 receptor (**B**, green), and DAPI (blue) immunofluorescence of VEH and GTN mice. (scale bars: 100 μm for overviews; 10 μm for zooms). Right: mean fluorescence intensity of Y1 receptor (**A**) and Y2 receptor (**B**) in the GTN group relative to the VEH group. *n* = 4 mice per group, six sections per mouse. Significance was assessed by a two-tailed unpaired Student’s t-test. All data are presented as the mean ± S.E.M. **P < 0.01

NPY acts via at least five different Y receptors in mice, of which the Y1 and Y2 receptors are the most abundant and intensively investigated in studies of nociception [53–55]. Importantly, the Y1 receptor has also been demonstrated to be involved in NPY-mediated anxiolytic effects [16, 56]. Hence, to obtain more insights into our results, we explored the protein expressions of the two major NPY receptors—Y1 and Y2 receptors in the MHb—by fluorescent staining. We first confirmed that there were Y1 and Y2 receptors expressed in the MHb (Fig. 6A, B). Secondly, the expression of the Y1 receptor was remarkably increased by GTN (Fig. 6A; *t*(2, 6) =  − 4.255, *P* = 0.005), while no difference was found in Y2 receptor expression between VEH and GTN mice (Fig. 6B; *t*(2, 6) =  − 1.037, *P* = 0.340). These findings suggest that the Y1 receptor may mediate the effects of NPY in the MHb.

**Fig. 7:**
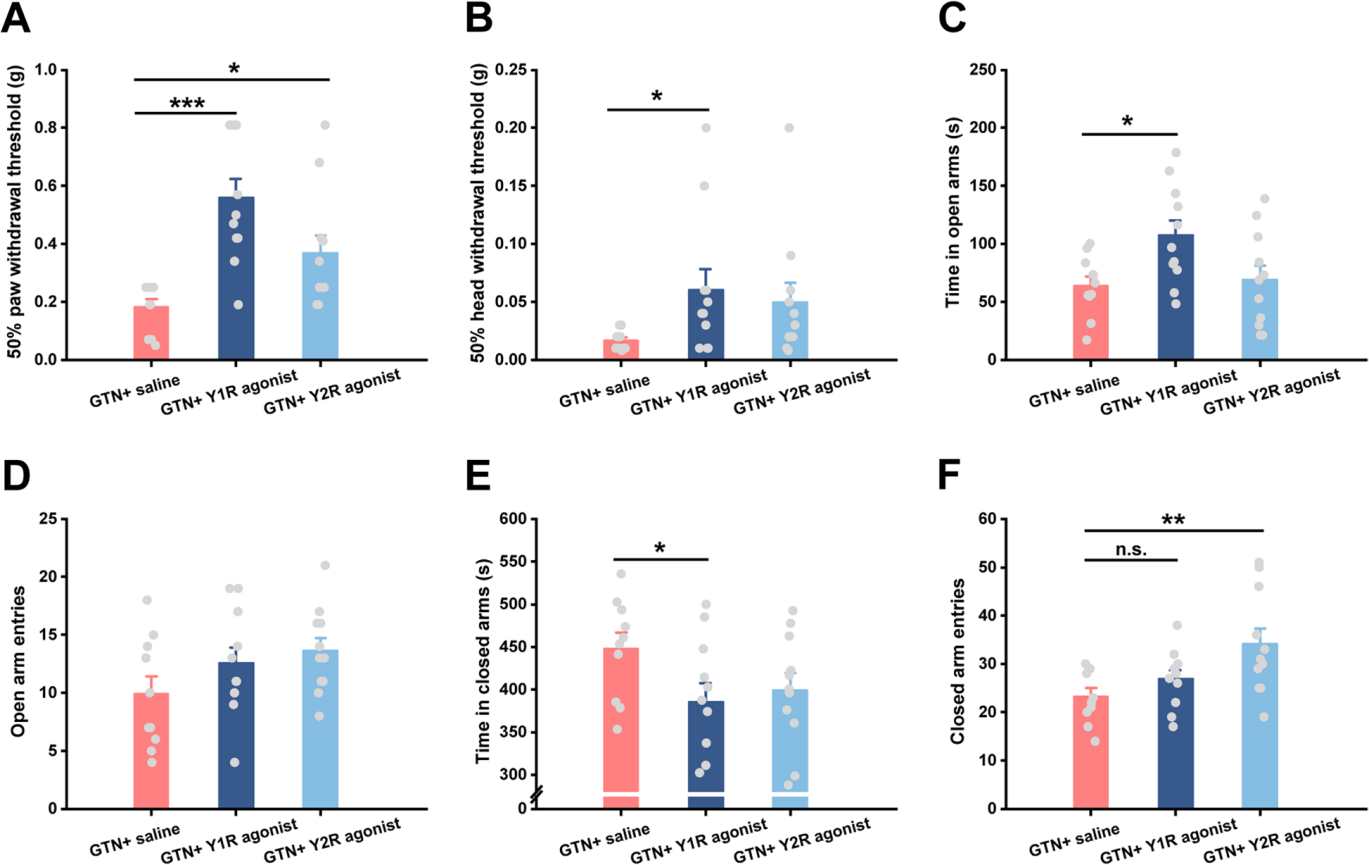
Microinjection of Y1 receptor agonist into the MHb alleviates GTN-induced allodynia and anxiety-like behaviors.** A** and **B** Effects of the Y1 receptor agonist or Y2 receptor agonist on the left hind paw (**A**) and head withdrawal thresholds (**B**) in the von Frey test at 1.5 h after VEH or GTN injection. **C**-**F** Effects of the Y1 receptor agonist or Y2 receptor agonist on time spent in the open arms (**C**), the number of open arm entries (**D**), time spent in the closed arms (**E**), and the number of closed-arm entries (**F**) during the EPM test at 4 h after VEH or GTN injection. GTN + saline, *n* = 10 mice; GTN + Y1 receptor agonist, *n* = 11 mice; GTN + Y2 receptor agonist, *n* = 11 mice. Significance was assessed by Kruskal–Wallis H test with Mann–Whitney U post hoc comparison between groups (A and B; *P < 0.05, ***P < 0.001) or by one-way ANOVA with post hoc comparison between groups (C-F; *P < 0.05, **P < 0.01). All data are presented as the mean ± S.E.M

Given the fact that the administration of NPY per se cannot discriminate which receptor pathways were activated, we then repeated the MHb microinjection experiment using Y1 and Y2 receptor agonists, respectively. Interestingly, as shown in Fig. 7A, activation of both Y1 and Y2 receptor signaling pathways in GTN mice significantly increased hind paw withdrawal thresholds, with a more profound effect by Y1 receptor agonist (*χ*^*2*^(2) = 15.045, *P* = 0.001; *U* = 6.000, *P* < 0.001, GTN + saline versus GTN + Y1R agonist; *U* = 22.000, *P* = 0.020, GTN + saline versus GTN + Y2R agonist). As for head allodynia, only GTN + Y1R agonist mice showed prominent alleviation of mechanical pain hypersensitivity compared to GTN + saline mice; we found no difference between GTN + Y2R agonist and GTN + saline mice in head pain hypersensitivity (Fig. 7B; *χ*^*2*^(2) = 6.341, *P* = 0.042; *U* = 22.000, *P* = 0.020, GTN + saline versus GTN + Y1R agonist; *U* = 28.000, *P* = 0.061, GTN + saline versus GTN + Y2R agonist). Moreover, activating the MHb Y1 receptor increased the time spent in open arms in the EPM test (*F*(2, 29) = 4.297, *P* = 0.023; post hoc comparison: *P* = 0.013), accompanied by less time in closed arms (Fig. 7C, E). Despite these differences, we did not observe any changes in the frequency entering open or closed arms (Fig. 7D, F). On the contrary, activating the Y2 receptor seemingly induced anxiety-like behaviors, as indicated by an increased number of closed-arm entries (Fig. 7F; *F*(2, 29) = 5.280, *P* = 0.011; post hoc comparison: *P* = 0.004). However, the time spent in open and closed arms and the frequency of open-arms entry were not affected by the Y2 receptor agonist (Fig. 7C–E). Finally, the motor abilities in EPM of all groups were similar, proved by the results of total distance travelled (Fig. S3). Together, these results indicate the MHb Y1 receptor is predominantly involved in relieving allodynia and anxiety-like behaviors induced by GTN.

## Discussion

Migraine is a common headache disorder with heterogeneous symptoms. In this study, we confirmed the allodynia, photophobia, and anxiety-like behaviors accompanied migraine. We also determined the timing of effects induced by acute GTN administration; these results provided the experimental basis for our research. Next, we generated a clear NPY expression map of the whole brain for the first time, and found considerable changes in NPY levels in select brain areas in GTN mice. These results will undoubtedly inform future studies. Finally, we initiatively identified the key role of MHb NPY in the alleviation of allodynia and anxiety-like behaviors through the Y1 receptor in the GTN-induced migraine model.

NPY is involved in migraine pathogenesis. Although Oliveira et al*.* [57] demonstrated that NPY reduced trigeminocervical complex neuronal firing through the Y1 receptor in a migraine rat model, NPY was applied systemically, meaning that it may potentially affect the entire neural network. Till date, the specific roles of NPY in migraine and how it functions after migraine onset remain unclear. Therefore, we must determine how NPY levels are altered following GTN administration. Unfortunately, studies that examined NPY changes in patients with migraine produced highly variable findings [18–20]. This variability may reflect trial design and study cohort differences. A recent preclinical study reported on dynamic changes in NPY using a rat model where migraine was induced by electrical stimulation of the trigeminal ganglia (TG). Here, NPY expression was significantly elevated immediately after stimulation in TG and at 12 h in the blood plasma. Further elevations were observed upon repeated stimulation [58], confirming the important role of NPY in migraine pathogenesis. Unlike previous research, in the current study, we determined whole-brain NPY expression levels using VISoR imaging and a well-accepted migraine mouse model. Our results revealed significant and early changes in NPY levels in the MHb, SPVO, NPC, PGRN, GU5, and SSp-n5 following GTN administration.

The MHb is a small, complex, and evolutionarily conserved structure located on the dorsal surface of the thalamus (epithalamus) and linking forebrain areas with midbrain monoaminergic centers [59, 60]. Although the MHb is somewhat neglected, there is increasing evidence of its important roles in pain [61], anxiety [62], depression [63], fear [64], and nicotine addiction behaviors [65]. The MHb acts as a major point of convergence where external stimuli are received, evaluated, and redirected to generate appropriate behaviors. Therefore, the fact that GTN mice showed photophobia, allodynia, and anxiety-like behaviors prompted us to further investigate reductions of NPY in the MHb using a mouse migraine model.

It has been demonstrated that NPY signaling inhibited pain in the PAG, PBN, trigeminal nucleus, and the dorsal horn of the spinal cord [57, 66–68]. The intracellular signaling mechanism in spine was previously elucidated. Briefly, NPY binds to the Gi-coupled Y1 receptor at key sites of pain transmission, inhibiting AC1 and intracellular signaling. Additionally, Y1R neurons hyperpolarize due to the opening of gated inwardly rectifying potassium channels (GIRK) and potassium influx following Gi activation. Thus, NPY exerts analgesic effects [55]. Here, our findings uncover that the MHb acts as an additional action site for NPY’s analgesic effects and uniquely inhibits GTN-induced migraine-like pain. Clinical and preclinical studies have revealed the involvement of the habenula in pain. Shelton and co-workers [69] showed bilateral habenula activation during noxious heat stimulation with high-field magnetic resonance imaging (fMRI); thus, the habenula appears involved in sensory processing. Deep brain stimulation in the habenula revealed pain to be one of the most common transient effects of increased voltage [70]. However, due to its small size, current MRI techniques (3 T scanners) do not allow us to distinguish MHb from LHb signals. Meanwhile, 7 T scanners seem capable of separating signals from MHb and LHb in ex-vivo brains, but not functionally in vivo [71, 72]. Because of this, rodent studies of MHb are more reliable. A recent preclinical study reported distinct activation patterns within the MHb under a neuropathic pain condition [73]; meanwhile, lesioning the MHb increased pain sensitivity [74]. Darcq et al*.* [61] reported that morphine acted through mu-opioid receptors directly on MHb neurons to produce analgesia. These data demonstrate that the MHb plays a role in direct analgesia through opioidergic systems. Given the high mu-opioid receptor density found in the MHb [75, 76], Y1 receptors in the MHb likely interact with opioidergic systems to produce analgesic effect. Alternatively, NPY signaling may also act as a novel neurotransmitter system and exert dependent nociception control. Several lines of evidence support the latter notion. Firstly, the intracellular mechanisms of signal transduction activated by NPY through Y1 receptors in spine have been reported to contribute to pain inhibition. Additionally, the presence of NPY and Y1 receptor has been proved in this structure by previous studies [77, 78] and our results, implying the direct analgesic effects of NPY signaling. Secondly, NPY Y1 and opioid receptors are Go/Gi-coupled receptors [61] and can activate the extracellular signal-regulated kinases (ERK1/2) [79]. Darcq et al*.* found that RSK2 signaling—a direct downstream effector of ERK1/2—contributed to acute morphine analgesia in the MHb. Thus, we speculate that NPY Y1 receptor-RSK2 signaling may directly mediate NPY’s analgesic effects. Thirdly, the MHb primarily projects to the interpeduncular nucleus (IPN), co-releasing acetylcholine and glutamate. As a downstream structure of the MHb-IPN axis, the dorsal nucleus raphe receives GABAergic projection from IPN [80]. Thereby, the NPY Y1 receptor signaling may activate descending antinociceptive pathways (brainstem modulatory systems) by reducing neuron activity in the MHb. Further studies are needed.

Furthermore, we found mild differences in the analgesic effects functioned by Y1 and Y2 receptors (Fig. 7A, B). Although cutaneous allodynia is associated with central sensitization and synaptic plasticity [81], there is a specific difference in the underlying mechanisms between head allodynia and paw allodynia. Sandkühler and Gruber-Schoffnegger [82] reviewed that allodynia in stimulated region is attributed to homosynaptic long-term potentiation (potentiation of the same synapses that were stimulated), whereas allodynia beyond the stimulated territory may be caused by heterosynaptic potentiation (potentiation of non-stimulated synapses on the same cell) occurs in both principal cells and interneurons of the dorsal horn in limb pain models. Similar processes may occur in the MHb in GTN-induced migraine model, which could explain our results that paw allodynia was alleviated by both Y1 and Y2 agonists, but head allodynia was suppressed only by Y1 agonists. The more abundant expression of Y1 receptor in the MHb (Fig. 6) and the different distribution patterns of the two receptors at the synapses may contribute to their functional differences in allodynia. The mechanisms will be further investigated.

In addition, there was a notable anxiolytic action of NPY and Y1R agonist, microinjected into the MHb, as evidenced by EPM. Previous works have demonstrated the anxiolytic effect of NPY acting at the Y1 receptor in other brain areas, including the amygdala, PAG, hippocampus, and lateral septum [83–85]. These effects are, in part, due to antagonism of stress-promoting signals such as corticotrophin-releasing factor [16, 86]. Although there are limited studies on the role of the MHb in anxiety in humans, many preclinical studies found the MHb to be a powerful modulator of anxiety-like behaviors. Early lesion and pharmacological studies have implicated the habenular pathway in anxiety-related behaviors in rodents [87–89]. These studies, however, did not identify the precise neural mechanisms underlying observed negative emotional behaviors due to technical limitations. Using the genetic tools, Yamaguchi et al*.* [62] demonstrated that TS → ventral MHb → IPN neural transmission was crucial for anxiety; ablation of this pathway reduced anxiety-related behaviors. Similarly, Pang et al*.* [90] expressed novel nAChR subunits selectively in MHb cholinergic neurons of adult mice, which was able to activate MHb cholinergic neurons, and found that the agonist of nAChR subunits increased anxiety levels. Thus, MHb cholinergic signaling appears important for modulating anxiety-like responses, with increased activity of MHb cholinergic neurons being associated with a heightened anxiety-like state, while the decreased activity of MHb cholinergic neurons being linked with decreased anxiety [91]. In line with these findings, in this study, we observed that NPY attenuated anxiety-like behaviors induced by GTN in the MHb; this at least partly attributed to the inhibitory effects of NPY on cholinergic neurons through the Y1 receptor. On the contrary, it is worth noting that some researches indicated that activation—but not inhibition—of MHb cholinergic signaling remarkably suppressed anxiety-like behaviors. For example, mice deprived of MHb neurons postnatally exhibited mildly increased anxiety levels [92]. Additionally, Vickstrom et al*.* [93] observed anxiolytic effects of endocannabinoid signaling in the MHb by depressing the synaptic GABA input in the ventral MHb from the medial septum and nucleus of the diagonal band. Such discrepancies may be derived from the different signaling pathways, animal models, and research methods used in these studies, as any of these factors can produce distinct behavioral manifestations. By contrast, we found that Y2 receptor activation mildly increased anxiety-like behaviors, similar to previous reports on the anxiogenic effects of NPY through Y2 receptors in the amygdala and lateral ventricles [94–96]. These results suggested that Y1 and Y2 receptors are expressed within different cell types and subtypes. The receptors may feature different neural circuits with potentially distinct functions. Furthermore, the responsiveness of NPY receptors can change under different conditions. Thorsell et al*.* [97] have demonstrated that overexpression of NPY in hippocampus of rats downregulated Y1 receptor binding but not Y2 receptor binding, suggesting subtype-specific functional changes of NPY receptors following NPY overexpression. In the present study, we surprisingly revealed that microinjection of NPY in VEH mice induced anxiety. This phenomenon could indicate that the Y1 receptor activity was down-regulated by NPY, and that Y2 receptor played a dominantly anxiogenic role in turn. After microinjecting NPY in GTN mice, the Y1 receptor mediated anxiolytic effects predominantly due to the upregulation of Y1 receptor expression by GTN (Fig. 6A). However, given that the precise mechanisms remain unclear, we will continue to pursue this matter in follow-up research.

Photophobia is one of the most common symptoms of migraine, and the underlying mechanism is uncertain. The anatomical structures of the potential neural circuits involved in the interaction between visual and pain pathways include the retina, olivary pretectal nucleus, superior salivatory nucleus, pterygopalatine ganglion, trigeminal afferents, trigeminal subnucleus caudalis, thalamus, hypothalamus, and cortex [98, 99]. Calcitonin gene-related peptide and pituitary cyclase-activating polypeptide are two important neuropeptides within photophobia circuits [100]. To date, no evidence has been found for the participation of MHb or NPY in photophobia, which is consistent with our findings that administration of NPY to MHb did not affect GTN-induced photophobia.

## Conclusions

To the best of our knowledge, this is the first report of the analgesic and anxiolytic effects of NPY partly through Y1 receptors when microinjected into the MHb, using a GTN-induced migraine mouse model. These findings offer novel evidence for further development of therapeutic drugs. NPY targeting within the MHb might emerge as a new and effective migraine treatment. However, one limitation of the current study is the lack of evidence regarding Y1 receptor involvement using receptor antagonists or AAV-based RNA interference. Moreover, further studies will be needed to examine other potentially implicated brain regions, including the SPVO, NPC, PGRN, GU5, and SSp-n5, and determine how the NPY system contributes to migraine symptoms.

## Supplementary Information

**Additional file 1:** **Figure S1.** The total distance travelled during the EPM testat 2 h (*n* = 8 mice per group), 4 h (*n* = 9 mice per group), and 24 h (*n* = 9 mice per group) after VEH or GTN injection. (Related to Fig. 2) Significance was assessed by two-tailed unpaired Student’s t-test. All data are presented as the mean ± S.E.M. **Figure S2.** Effects of NPY or saline on the total distance travelled during the EPM test at 4 h after VEH or GTN injection. VEH + saline, *n* = 11 mice; GTN + saline, *n* = 10 mice; VEH + NPY, *n* = 11 mice; GTN + NPY, *n* = 11 mice. (Related to Fig. 5) Significance was assessed by two-way ANOVA. All data are presented as the mean± S.E.M. **Figure S3.** Effects of Y1 receptor agonist or Y2 receptor agonist on the total distance travelled during the EPM test at 4 h after VEH or GTN injection. GTN + saline, *n* = 10 mice; GTN + Y1 receptor agonist, *n* =11 mice; GTN + Y2 receptor agonist, *n* = 11 mice. (Related to Fig. 7) Significance was assessed by one-way ANOVA. All data are presented as the mean ± S.E.M. **Figure S4.** The representative images of histological sites of cannula placements in the MHb. Blue, 0.25% Evans Blue. Scale bars, 1000 μm (A) and 200 μm (B). The yellow contour delineates the borders of the MHb.

**Additional file 2: Table S1.** The quantitative number of NPY neurons in a maximum of 836 brain regions in VEH and GTN mice.

## Data Availability

All data, reagents, resources, and protocols are available from the corresponding author upon reasonable requests.

## Abbreviations

EPM: Elevated plus maze
GTN: Glyceryl trinitrate
GU5: Gustatory areas layer 5
HMS: Hydrogel monomer solution
IPN: Interpeduncular nucleus
LP: Lateral posterior nucleus of the thalamus
MHb: Medial habenula
NPC: Nucleus of the posterior commissure
NPY: Neuropeptide Y
NTS: Nucleus of the solitary tract
PFA: Paraformaldehyde
PGRN: Paragigantocellular reticular nucleus
SPVC: Spinal nucleus of the trigeminal caudal part
SPVO: Spinal nucleus of the trigeminal oral part
SSp: Primary somatosensory area
SSp-n5: Primary somatosensory area nose layer 5
SSs: Supplemental somatosensory area
VEH: Vehicle
VISoR: Volumetric imaging with synchronized on-the-fly-scan and readout
VPM: Ventral posteromedial nucleus of the thalamus
