# Supplementary material for: Neuropeptide Y in the medial habenula alleviates migraine-like behaviors through the Y1 receptor
Source: J Headache Pain. 2023 May 25;24(1):61. doi: 10.1186/s10194-023-01596-z (PMC10209951; doi:10.1186/s10194-023-01596-z)
Supplement: Supplementary file 1 — Additional file 1: Figure S1. The total distance travelled during the EPM testat 2 h (n = 8 mice per group), 4 h (n = 9 mice per group), and 24 h (n = 9 mice per group) after VEH or GTN injection. (Related to Fig. 2) Significance was assessed by two-tailed unpaired Student’s t-test. All data are presented as the mean ± S.E.M. Figure S2. Effects of NPY or saline on the total distance travelled during the EPM test at 4 h after VEH or GTN injection. VEH + saline, n = 11 mice; GTN + saline, n = 10 mice; VEH + NPY, n = 11 mice; GTN + NPY, n = 11 mice. (Related to Fig. 5) Significance was assessed by two-way ANOVA. All data are presented as the mean± S.E.M. Figure S3. Effects of Y1 receptor agonist or Y2 receptor agonist on the total distance travelled during the EPM test at 4 h after VEH or GTN injection. GTN + saline, n = 10 mice; GTN + Y1 receptor agonist, n =11 mice; GTN + Y2 receptor agonist, n = 11 mice. (Related to Fig. 7) Significance was assessed by one-way ANOVA. All data are presented as the mean ± S.E.M. Figure S4. The representative images of histological sites of cannula placements in the MHb. Blue, 0.25% Evans Blue. Scale bars, 1000 μm (A) and 200 μm (B). The yellow contour delineates the borders of the MHb. [file 10194_2023_1596_MOESM1_ESM.pdf]

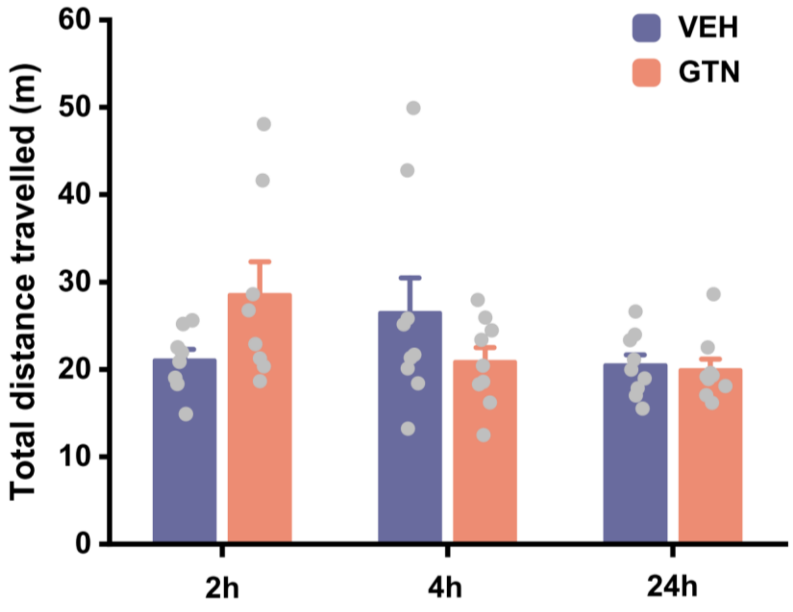

Figure S1. The total distance travelled during the EPM test at 2 h (n = 8 mice per group), 4 h (n = 9 mice per group), and 24 h (n = 9 mice per group) after VEH or GTN injection. (Related to Fig. 2) Significance was assessed by two-tailed unpaired Student's t-test. All data are presented as the mean ± S.E.M.

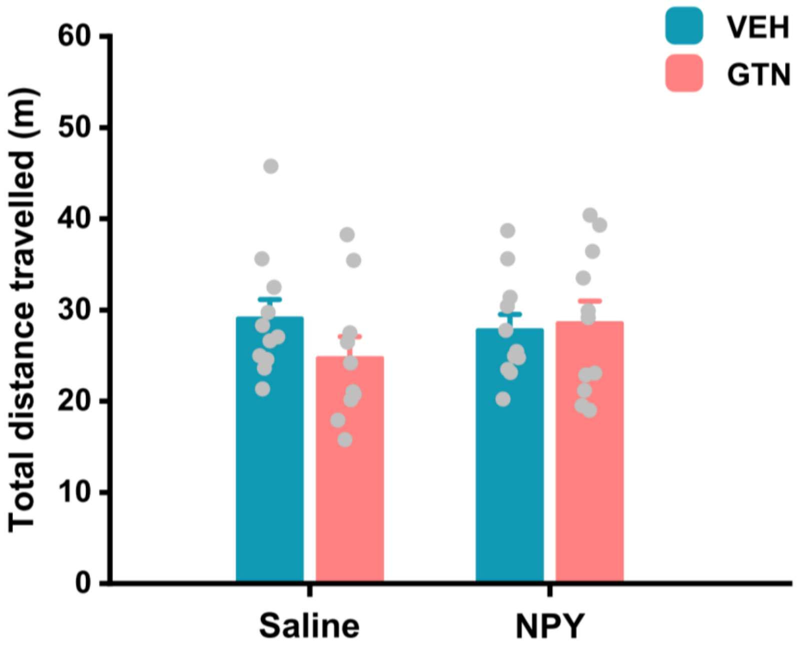

Figure S2. Effects of NPY or saline on the total distance travelled during the EPM test at 4 h after VEH or GTN injection. VEH + saline, n = 11 mice; GTN + saline, n = 10 mice; VEH + NPY, n = 11 mice; GTN + NPY, n = 11 mice. (Related to Fig. 5) Significance was assessed by two-way ANOVA. All data are presented as the mean ± S.E.M.

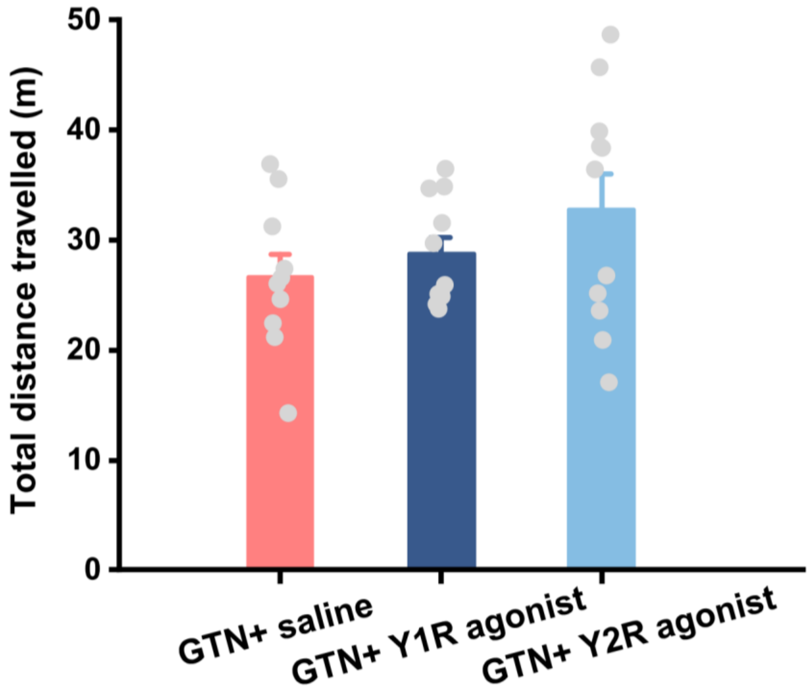

Figure S3. Effects of Y1 receptor agonist or Y2 receptor agonist on the total distance travelled during the EPM test at 4 h after VEH or GTN injection. GTN + saline, n = 10 mice; GTN + Y1 receptor agonist, n = 11 mice; GTN + Y2 receptor agonist, n = 11 mice. (Related to Fig. 7) Significance was assessed by one-way ANOVA. All data are presented as the mean ± S.E.M.

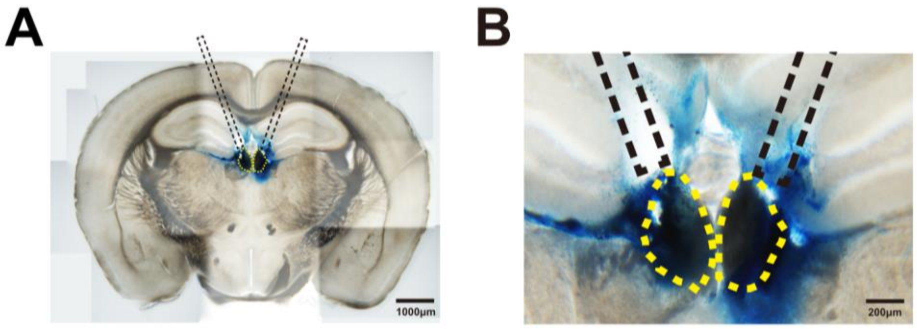

Figure S4. The representative images of histological sites of cannula placements in the MHb. Blue, 0.25% Evans Blue. Scale bars, 1000 μm (**A**) and 200 μm (**B**). The yellow contour delineates the borders of the MHb.
